# FAM72 serves as a biomarker of poor prognosis in human lung adenocarcinoma

**DOI:** 10.18632/aging.202625

**Published:** 2021-03-03

**Authors:** Yilin Yu, Zhiping Wang, Qunhao Zheng, Jiancheng Li

**Affiliations:** 1 Fujian Medical University Cancer Hospital, Fujian Cancer Hospital, Fuzhou, Fujian, China

**Keywords:** FAM72 family, lung adenocarcinoma, The Cancer Genome Atlas, prognosis, immune infiltration

## Abstract

FAM72A–D promote the self-renewal of neural progenitor cells. There is accumulating evidence that FAM72 promotes tumorigenicity. However, its effects in lung adenocarcinoma (LUAD) have not been determined. Thus, we evaluated the prognostic value of *FAM72A–D* in LUAD using bioinformatics approaches. In particular, we evaluated the relationship between FAM72 and LUAD using a wide range of databases and analysis tools, including TCGA, GEO, GEPIA, Metascape, cBioPortal, and MethSurv. Compared with its expression in normal lung tissues, FAM72 expression was significantly increased in LUAD tissues. A univariate Cox analysis showed that high FAM72 expression levels were correlated with a poor OS in LUAD. Additionally, FAM72 expression was independently associated with OS through a multivariate Cox analysis. GO and GSEA revealed enrichment in mitotic nuclear division and cell cycle. Moreover, high FAM72 expression was associated with poor survival. An analysis of immune infiltration showed that FAM72 is correlated with immune cell infiltration. Finally, we found that the methylation level was associated with prognosis in patients with LUAD. In summary, these results indicate that FAM72 is a potential molecular marker for poor prognosis in LUAD and provide additional insight for the development of therapies and prognostic markers.

## INTRODUCTION

Lung adenocarcinoma (LUAD) and lung squamous cell carcinoma are the two main types of non-small cell lung cancer (NSCLC) [1]. LUAD is the most common histological type of NSCLC [2]. Lung cancer has become the second most common cancer in both men and women. It accounts for 25% of all cancer-related deaths. During 2007–2013, the 5-year relative survival rate of lung cancer was only 18%. It is usually diagnosed at a relatively advanced stage [3, 4]. Currently, the treatment for LUAD mainly includes surgical resection, chemotherapy, radiotherapy, and molecular targeted therapy. However, compared with the steadily increasing survival rates for many cancers, the overall survival (OS) for lung cancer has not improved substantially, primarily owing to the lack of effective therapeutic targets [5]. The most important and only curable treatment for LUAD is surgical resection. However, once distant metastasis occurs, LUAD cannot be cured through surgery. Therefore, there is an urgent need to identify novel molecular mechanisms and effective therapeutic targets for LUAD.

*FAM72A–D* (Family with sequence similarity 72 member A–D) is composed of four human-specific paralogs (A–D). *FAM72* is a protein-coding neural stem cell-specific gene [6, 7]. However, under certain conditions, *FAM72* may lead to post-mitotic neuronal death and may promote the occurrence and development of cancers in tissues other than neuronal tissues [6, 8, 9], including breast cancer, prostate cancer, and glioblastoma [6, 10, 11]. FAM72 is associated with cancer cell division, proliferation, and differentiation. Additionally, high levels of FAM72 are associated with hypomethylation and affect the prognosis in various cancers [11, 12]. These results suggest that FAM72 is a prognostic biomarker in cancer. However, it has not been established as a prognostic factor in LUAD, and its relationship with LUAD has not been reported to date.

In this study, we performed the first analysis of the functions of FAM72 in LUAD. In particular, The Cancer Genome Atlas (TCGA) was utilized to analyze the expression of FAM72A–D and its clinical-prognostic value in LUAD. We validated the prognostic value in four independent datasets from the Gene Expression Omnibus (GEO) database. We first analyzed the expression differences of FAM72 in patients with LUAD between tumor tissue and normal tissue and then explored correlations between expression levels and clinicopathological parameters based on univariate and multivariate Cox regression models. We further developed a nomogram to predict prognosis and analyzed its predictive performance. To gain a more in-depth understanding of the biological mechanisms underlying the effects of FAM72, we performed a Gene Ontology (GO) analysis and a gene set enrichment analysis (GSEA). Finally, we examined the correlations between FAM72 and mutations, immune infiltration, and methylation and comprehensively explored the link between FAM72 and tumorigenesis.

## RESULTS

### Clinical characteristics

Data (shown in Table 1) were collected from TCGA, including gene expression data and clinical data. Patient characteristics, including age, gender, pack-years, race, tumor site, epithelial growth factor receptor (EGFR) status, anaplastic lymphoma kinase (ALK) status, kirsten rat sarcoma viral oncogene (KRAS) status, TNM stage, pathological stage, and gene expression, were collected.

**Table 1:** TCGA lung adenocarcinoma patient characteristics.

**Clinical characteristics**		**Total (497)**	**Percentage (%)**
Gender			
	male	228	45.9
	female	269	54.1
Age			
	<=70 years old	327	65.8
	> 70 years old	160	32.2
Number pack years smoked			
	<40	167	33.6
	>=40	174	35
Race			
	white	384	77.3
	other	113	22.7
Tumor site			
	upper lobe	291	58.6
	other	206	41.4
EGFR status			
	mut	79	15.9
	wt	190	38.2
ALK status			
	mut	33	6.6
	wt	206	41.4
KRAS status			
	mut	61	12.3
	wt	244	49.1
T stage			
	T1	166	33.4
	T2	267	53.7
	T3	43	8.7
	T4	18	3.6
N stage			
	N0	321	64.6
	N1	94	18.9
	N2	69	13.9
	N3	2	0.4
M stage			
	M0	331	66.6
	M1	24	4.8
TNM stage			
	stage I	267	53.7
	stage II	118	23.7
	stage III	80	16.1
	stage IV	25	5
Vital status			
	dead	180	36.2
	alive	317	63.8
FAM72A expression			
	low	248	49.9
	high	249	50.1
FAM72B expression			
	low	248	49.9
	high	249	50.1
FAM72C expression			
	low	248	49.9
	high	249	50.1
FAM72D expression			
	low	248	49.9
	high	249	50.1

### Expression status of FAM72 in LUAD tissues

**Figure 1:**
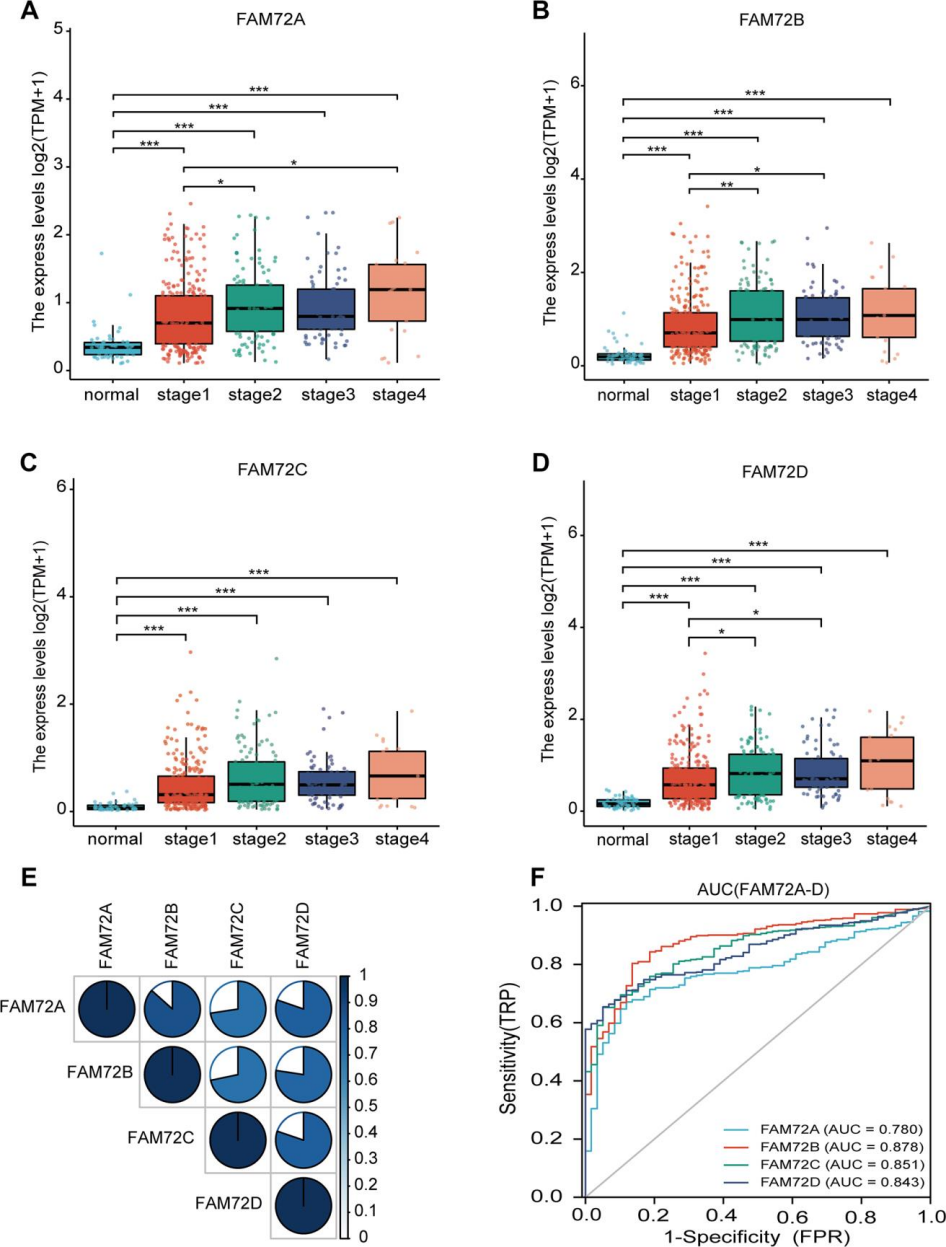
**FAM72A-D expression levels in LUAD from TCGA data.** (**A**–**D**) The expression levels of FAM72A-D in LUAD and normal tissue; (**E**) The correlation between FAM72A-D members; (**F**) Receiver operating characteristic analysis (ROC) of FAM72A-D in LUAD. (*P < 0.05, **P < 0.01, ***P < 0.001).

As evaluated by the Wilcoxon rank-sum test, the expression of FAM72A–D was significantly higher in LUAD tissues than in normal tissues (*P* < 0.001) (Figure 1A–1D). FAM72A–D was significantly overexpressed in LUAD (*P* < 0.001), revealing that the expression of FAM72A–D is associated with lung carcinogenesis. As shown in Figure 1E, there were significant correlations between levels of FAM72A–D genes. The AUC values for FAM72A–D were 0.780 (CI: 0.733−0.826), 0.878 (CI: 0.840−0.917), 0.851(CI: 0.813−0.890), and 0.843 (CI: 0.807−0.880), respectively (Figure 1F).

### Association between FAM72 and survival

**Figure 2:**
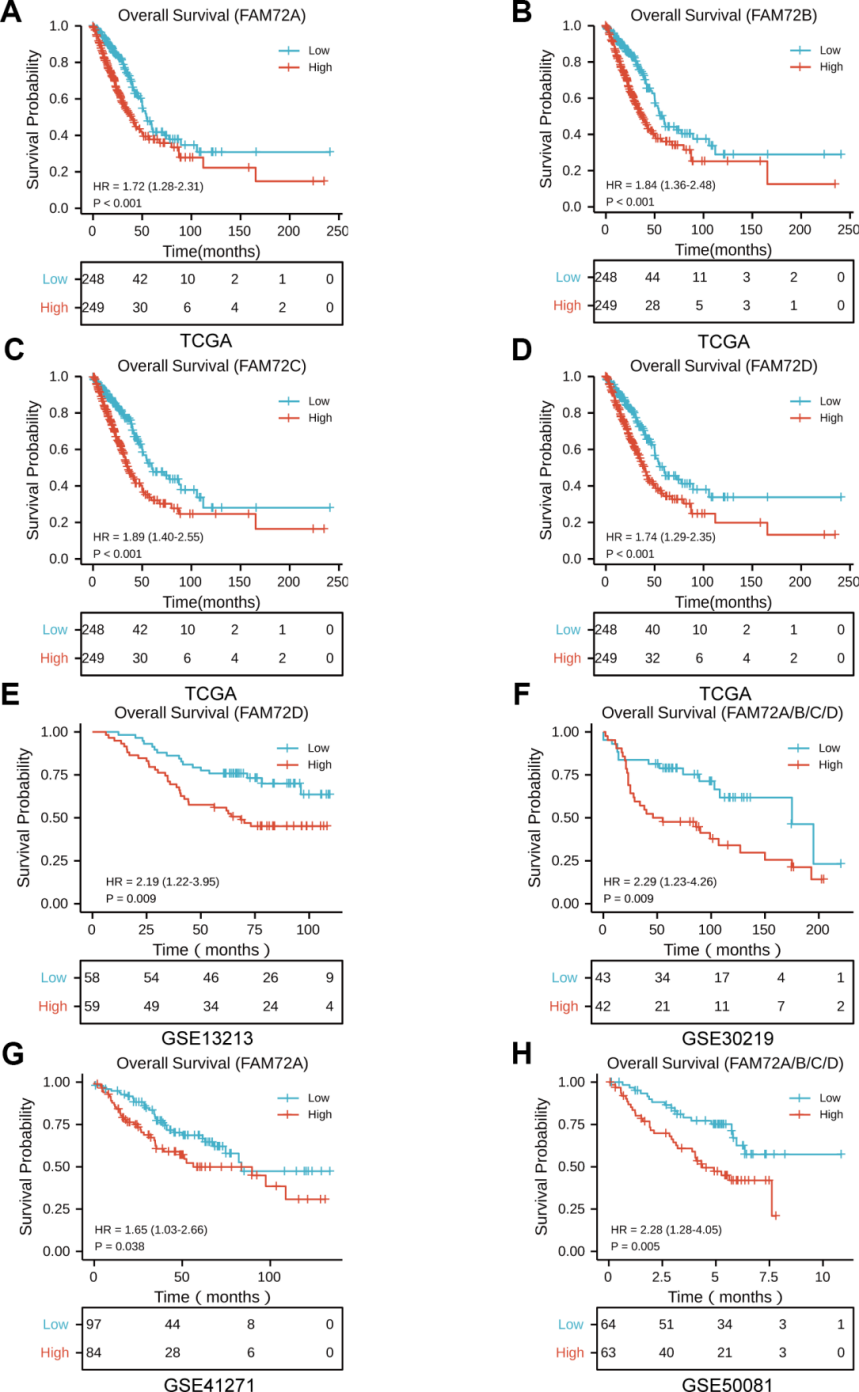
**The prognostic value of FAM72A-D expression in LUAD.** (**A**–**D**) Survival curves of OS from TCGA data (n = 497); (**E**–**H**) Survival curves of OS from GSE 13213, GSE30219, GSE41271 and GSE50081 data (n = 117, 85, 181, and 127, respectively).

OS was significantly reduced in patients with high FAM72A–D expression than in patients with low FAM72A–D expression (*P* ≤ 0.001) (Figure 2A–2D). To verify the association between FAM72A–D expression and OS, we used the GSE13213, GSE30219, GSE41271, and GSE50081 datasets from the GEO database. In these datasets, OS was significantly reduced in patients with high FAM72 expression than in those with low FAM72 expression (*P* = 0.009, 0.009, 0.038, and 0.005, respectively) (Figure 2E–2H). We next used a univariate Cox regression model to analyze prognostic factors in LUAD (Table 2). In this analysis, high FAM72 expression levels were correlated with a worse OS. We then performed a multivariate analysis with the Cox regression model. Owing to missing data exceeding 20%, the M stage was not included in this analysis. The results indicated that FAM72A–D expression (all *P* < 0.01), age, and pathological stage are independently associated with OS. These findings demonstrated that increased FAM72 expression is correlated with a poor OS in LUAD.

**Table 2:** Univariate(a) and multivariate(b) Cox regression model of prognosis for FAM72A-D in patients with lung adenocarcinoma.

**Clinicopathologic variable**	**Total(N)**	**HR(95% CI)**	**p-value**
FAM72A			
a.			
Gender(Male vs. Female)	497	0.954(0.711~1.279)	0.752
Age(>70 vs. <=70)	487	1.464(1.081~1.982)	0.014
Number pack years smoked(>40 vs. <=40)	341	1.026(0.714~1.475)	0.888
Race(Other vs. White)	497	1.265(0.797~2.008)	0.061
Tumor site(Upper lobe vs. Other)	497	1.156(0.862~1.552)	0.333
EGFR status(Mut vs. Wt)	266	1.265(0.797~2.008)	0.319
ALK status(Mut vs. Wt)	236	1.713(0.938~3.128)	0.080
KRAS status(Mut vs. Wt)	302	1.257(0.778~2.032)	0.351
T stage(T2/T3/T4 vs. T1)	494	1.678(1.187~2.373)	0.003
M stage(M1 vs. M0)	355	2.129(1.243~3.648)	0.006
Pathologic stage(StageII/Stage III/Stage IV vs. Stage I)	490	2.629(1.924~3.591)	<0.001
FAM72A (High vs. Low)	497	1.719(1.277~2.313)	<0.001
b.			
Age(>70 vs. <=70)		1.564(1.139~2.148)	0.006
T stage(T2/T3/T4 vs. T1)		1.232(0.856~1.774)	0.260
N stage(N2/N3 vs. N0/N1)		1.169(0.782~1.746)	0.447
FAM72A (High vs. Low)		1.714(1.250~2.351)	0.001
FAM72B			
a.			
Gender(Male vs. Female)	497	0.954(0.711~1.279)	0.752
Age(>70 vs. <=70)	487	1.464(1.081~1.982)	0.014
Number pack years smoked(>40 vs. <=40)	341	1.026(0.714~1.475)	0.888
Race(Other vs. White)	497	1.265(0.797~2.008)	0.061
Tumor site(Upper lobe vs. Other)	497	1.156(0.862~1.552)	0.333
EGFR status(Mut vs. Wt)	266	1.265(0.797~2.008)	0.319
ALK status(Mut vs. Wt)	236	1.713(0.938~3.128)	0.080
KRAS status(Mut vs. Wt)	302	1.257(0.778~2.032)	0.351
T stage(T2/T3/T4 vs. T1)	494	1.678(1.187~2.373)	0.003
M stage(M1 vs. M0)	355	2.129(1.243~3.648)	0.006
Pathologic stage(StageII/Stage III/Stage IV vs. Stage I)	490	2.629(1.924~3.591)	<0.001
b.			
Age(>70 vs. <=70)		1.595(1.160~2.193)	0.004
T stage(T2/T3/T4 vs. T1)		1.257(0.875~1.807)	0.216
N stage(N2/N3 vs. N0/N1)		1.131(0.755~1.694)	0.551
Pathologic stage(StageII/Stage III/Stage IV vs. Stage I)		2.326(1.631~3.318)	0.001
FAM72B (High vs. Low)		1.776(1.289~2.446)	<0.001
FAM72C			
a.			
Gender(Male vs. Female)	497	0.954(0.711~1.279)	0.752
Age(>70 vs. <=70)	487	1.464(1.081~1.982)	0.014
Number pack years smoked(>40 vs. <=40)	341	1.026(0.714~1.475)	0.888
Race(Other vs. White)	497	1.265(0.797~2.008)	0.061
Tumor site(Upper lobe vs. Other)	497	1.156(0.862~1.552)	0.333
EGFR status(Mut vs. Wt)	266	1.265(0.797~2.008)	0.319
ALK status(Mut vs. Wt)	236	1.713(0.938~3.128)	0.080
KRAS status(Mut vs. Wt)	302	1.257(0.778~2.032)	0.351
T stage(T2/T3/T4 vs. T1)	494	1.678(1.187~2.373)	0.003
N stage(N2/N3 vs. N0/N1)	486	2.274(1.589~3.255)	<0.001
M stage(M1 vs. M0)	355	2.129(1.243~3.648)	0.006
Pathologic stage(StageII/Stage III/Stage IV vs. Stage I)	490	2.629(1.924~3.591)	<0.001
FAM72C (High vs. Low)	497	1.907(1.414~2.572)	<0.001
b.			
Age(>70 vs. <=70)		1.507(1.100~2.064)	0.011
T stage(T2/T3/T4 vs. T1)		1.226(0.852~1.764)	0.272
N stage(N2/N3 vs. N0/N1)		1.220(0.815~1.825)	0.334
Pathologic stage(StageII/Stage III/Stage IV vs. Stage I)		2.238(1.566~3.197)	<0.001
FAM72C (High vs. Low)		1.796(1.310~2.462)	<0.001
FAM72D			
a.			
Gender(Male vs. Female)	497	0.954(0.711~1.279)	0.752
Age(>70 vs. <=70)	487	1.464(1.081~1.982)	0.014
Number pack years smoked(>40 vs. <=40)	341	1.026(0.714~1.475)	0.888
Race(Other vs. White)	497	1.265(0.797~2.008)	0.061
Tumor site(Upper lobe vs. Other)	497	1.156(0.862~1.552)	0.333
EGFR status(Mut vs. Wt)	266	1.265(0.797~2.008)	0.319
ALK status(Mut vs. Wt)	236	1.713(0.938~3.128)	0.080
KRAS status(Mut vs. Wt)	302	1.257(0.778~2.032)	0.351
T stage(T2/T3/T4 vs. T1)	494	1.678(1.187~2.373)	0.003
N stage(N2/N3 vs. N0/N1)	486	2.274(1.589~3.255)	<0.001
M stage(M1 vs. M0)	355	2.129(1.243~3.648)	0.006
Pathologic stage(StageII/Stage III/Stage IV vs. Stage I)	490	2.629(1.924~3.591)	<0.001
FAM72D (High vs. Low)	497	1.741(1.289~2.349)	<0.001
b.			
Age(>70 vs. <=70)		1.537(1.121~2.108)	0.008
T stage(T2/T3/T4 vs. T1)		1.225(0.851~1.764)	0.275
N stage(N2/N3 vs. N0/N1)		1.164(0.778~1.743)	0.459
Pathologic stage(StageII/Stage III/Stage IV vs. Stage I)		2.332(1.633~3.331)	<0.001
FAM72D (High vs. Low)		1.698(1.234~2.336)	0.001

### Development of a prognostic model based on FAM72 and clinicopathological factors

**Figure 3:**
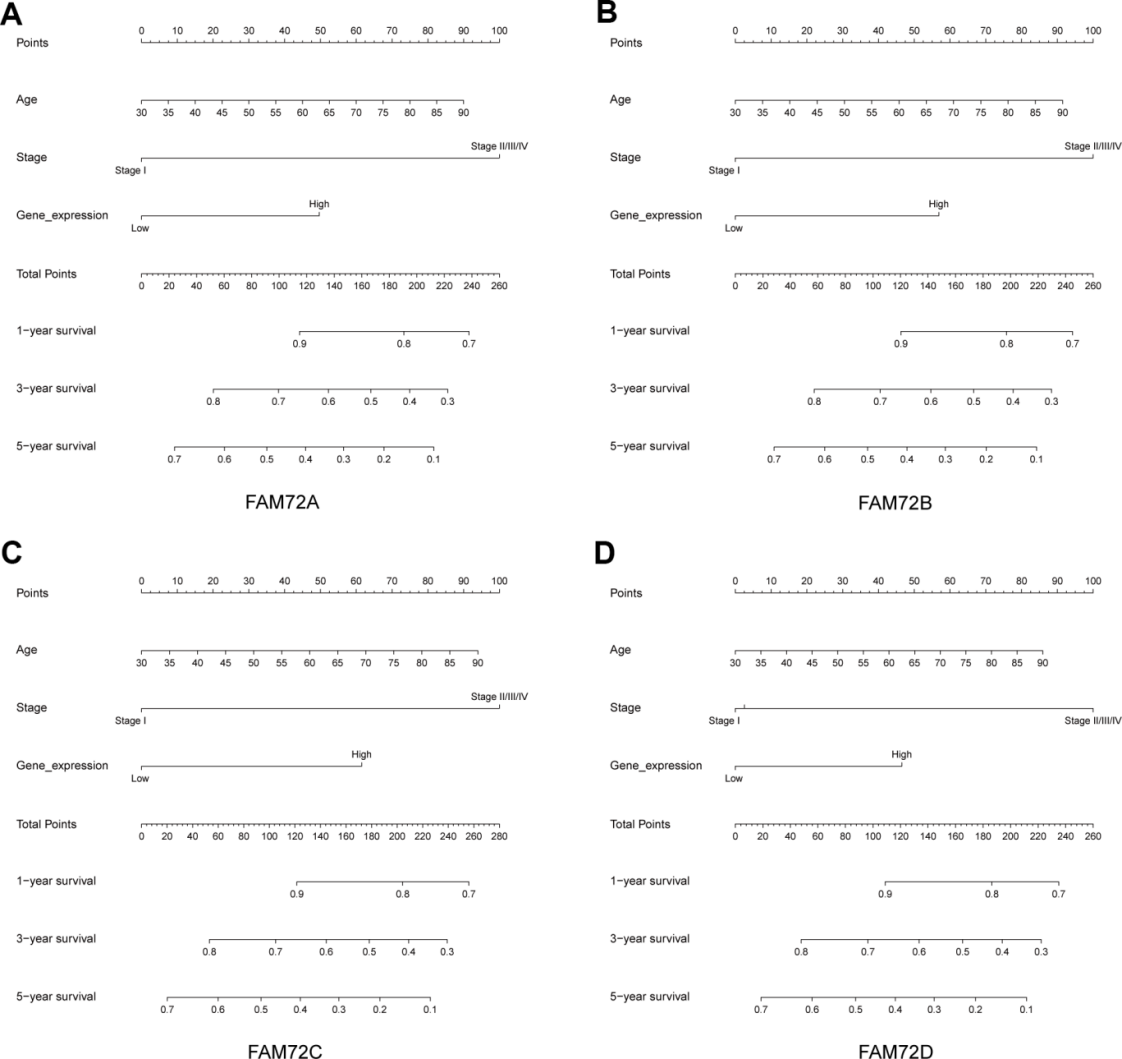
**Nomogram for predicting the probability of 1-, 3- and 5-year OS for LUAD patients.** (**A**–**D**) A nomogram that integrates FAM72A-D and other prognostic factors in LUAD from TCGA data.

**Figure 4:**
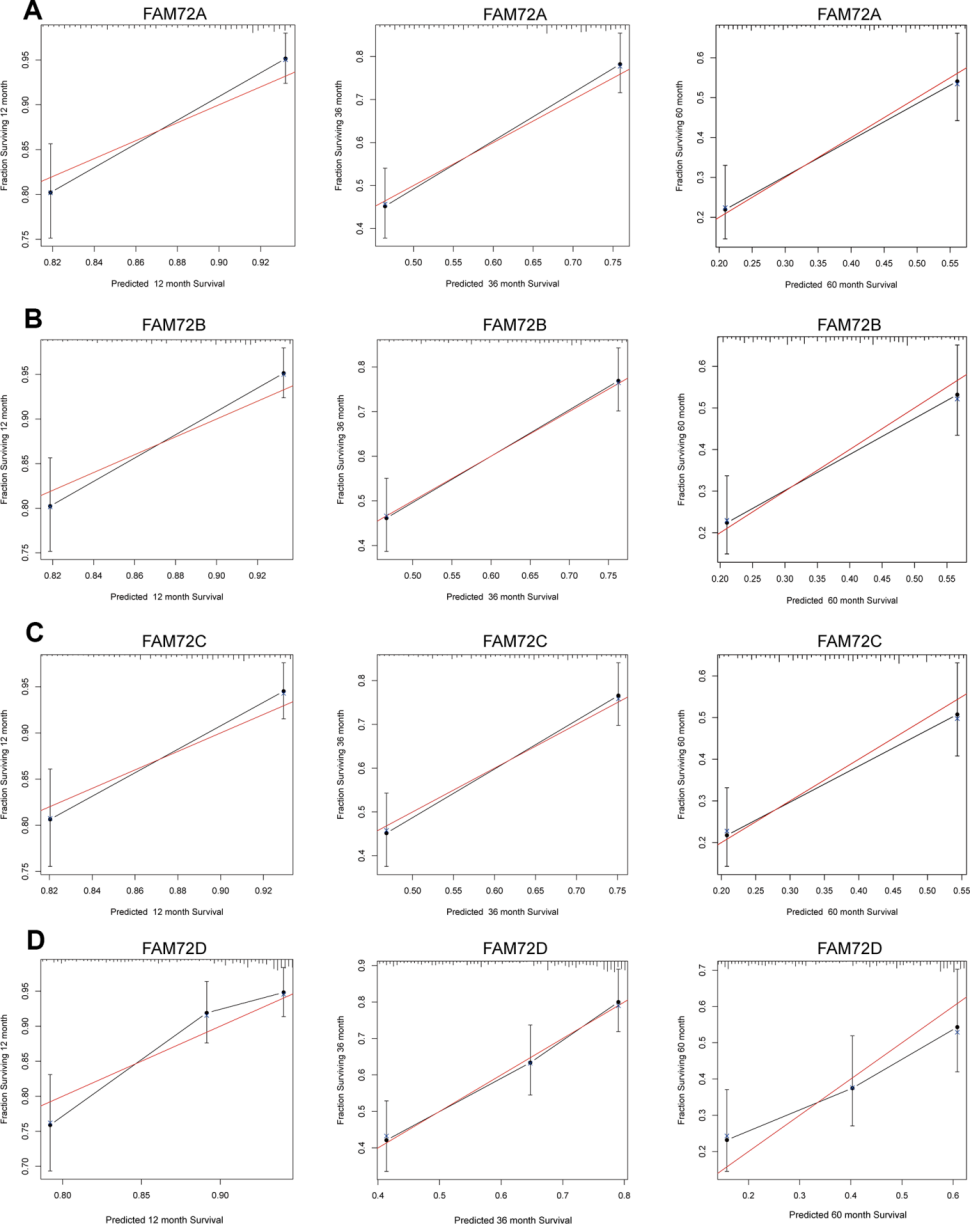
**Calibration curve for predicting the probability of 1-, 3- and 5-year OS for LUAD patients.** (**A**–**D**) The calibration curve of the nomogram in LUAD from TCGA data.

A nomogram integrating FAM72A–D expression and independent clinical risk factors (age and pathological stage) was constructed (Figure 3A–3D). A worse prognosis was represented by a higher total number of points on the nomogram. The C-index values were 0.7, 0.7, 0.69, and 0.68 for FAM72A–D based on 1000 bootstrap replicates. The deviation correction line in the calibration plot was close to the ideal curve (i.e., a 45° line), indicating that the prediction results are in good agreement with the observation results (Figure 4A–4D).

### FAM72-related functional enrichment analysis

**Figure 5:**
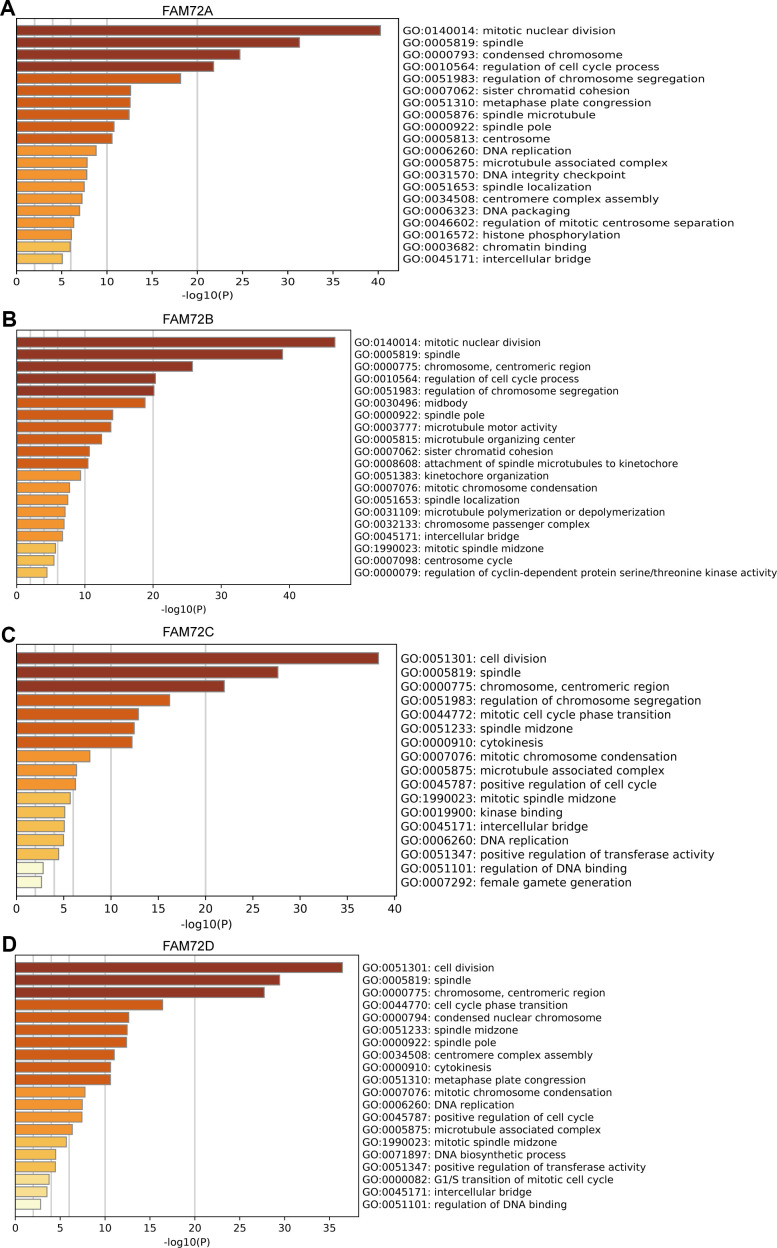
**Functional enrichment of FAM72A-D in LUAD.** (**A**–**D**) Gene ontology (GO) enrichment analysis of FAM72A-D and its co-expression genes in Metascape. The GO enriched terms are colored by *p*-value, where terms containing more genes tend to have more significant *p*-value.

**Figure 6:**
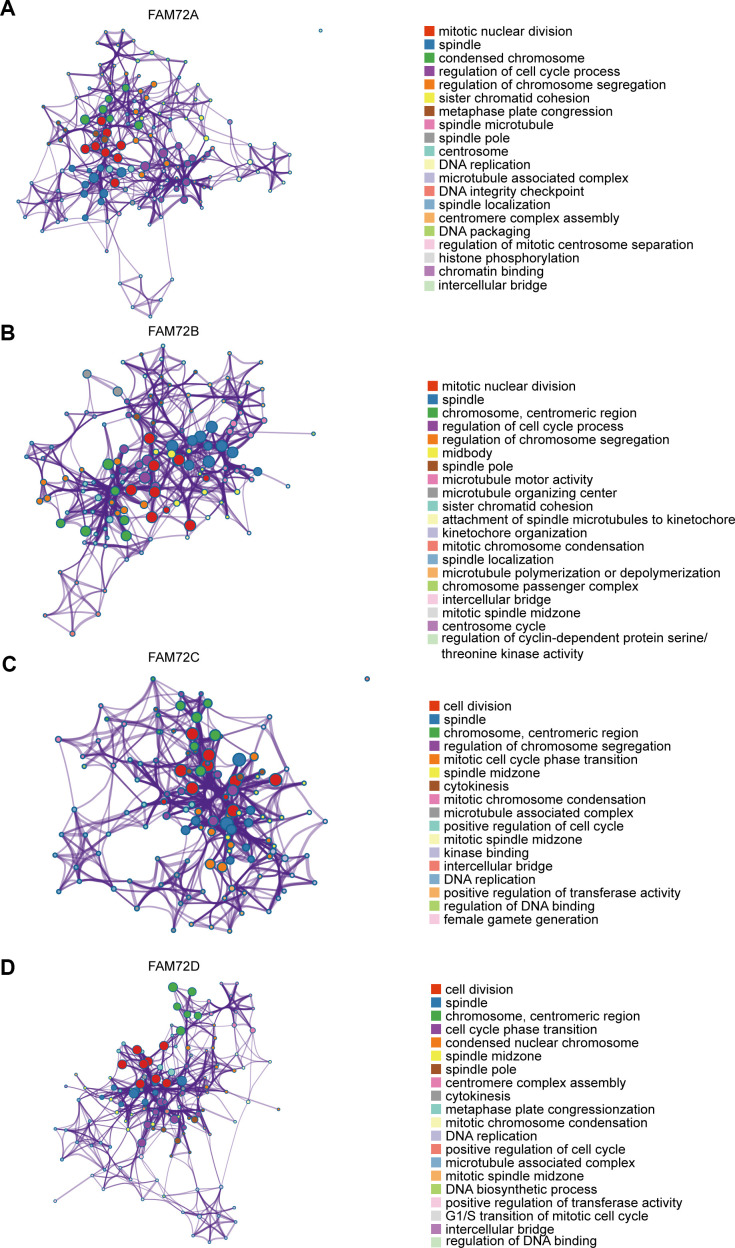
**An interactive network of the top enrichment terms.** (**A**–**D**) It is colored by cluster-ID. Distinct colors are various enrichment pathways of FAM72A-D correlated genes.

A GO enrichment analysis of genes with correlated expression revealed various overrepresented terms in the three main functional groups (Figure 5A–5D): cellular component, biological process, and molecular function. In the cellular component category, FAM72 and genes with similar expression patterns were mainly involved in mitotic nuclear division, spindle, cell cycle, and DNA replication. Figure 6A–6D shows an interactive network of the most highly enriched terms (colored by cluster-ID, where distinct colors indicate enriched pathways).

### FAM72-related signaling pathways obtained by GSEA

**Figure 7:**
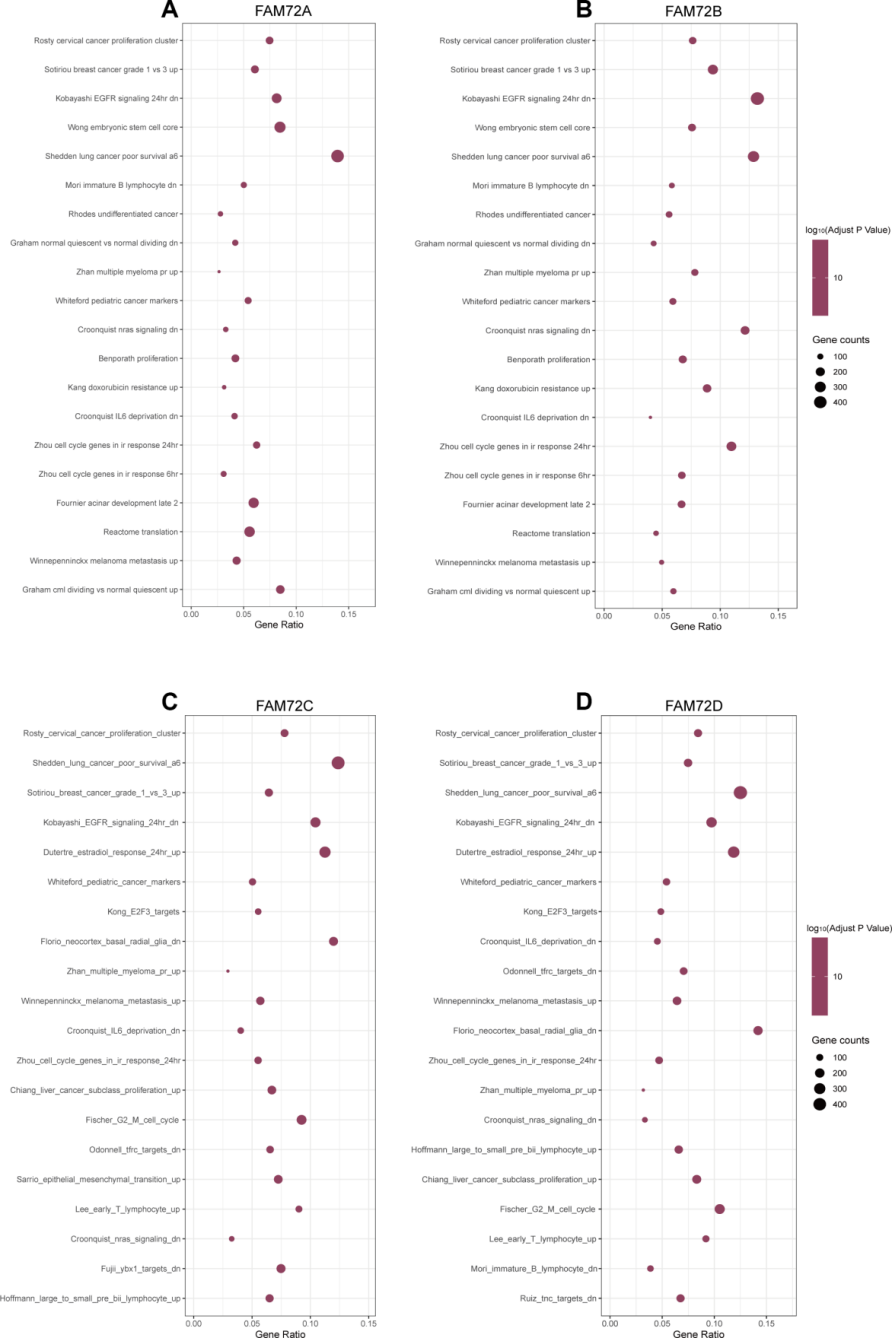
**The top 20 pathways were differentially enriched according to the level of NES in FAM72A-D related LUAD.** (**A**–**D**) The enrichment plot was obtained from the gene set enrichment analysis (GSEA).

**Figure 8:**
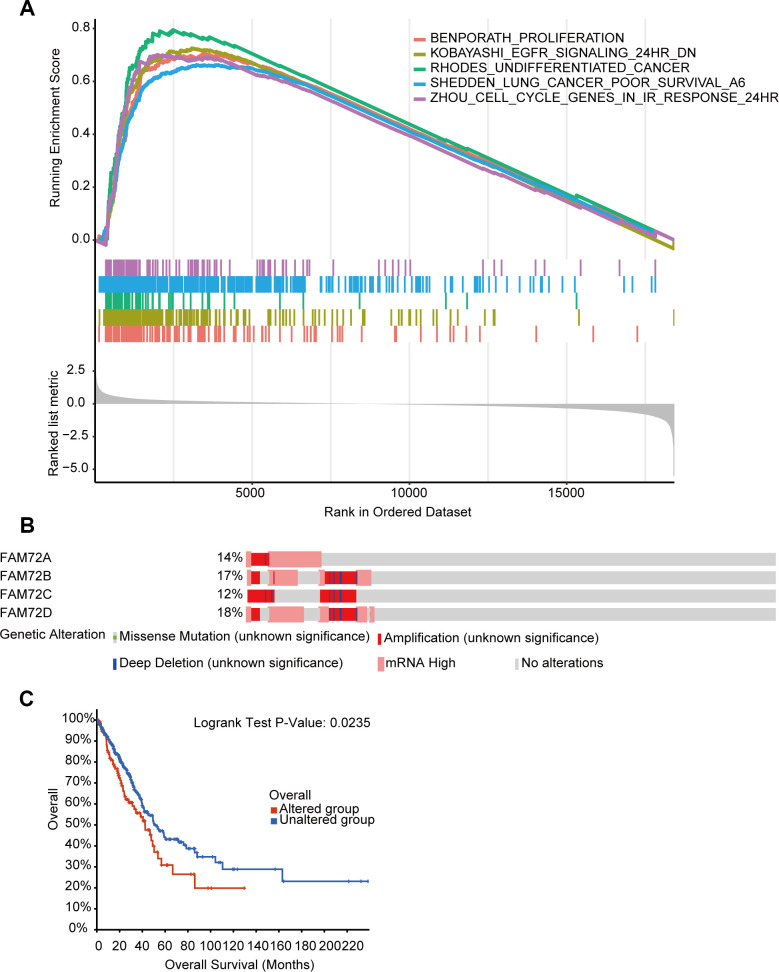
**The functional pathways and gene alteration of FAM72A-D in LUAD.** (**A**) Several pathways were enriched in FAM72A-D related LUAD, including the proliferation, EGFR signaling, undifferentiated cancer, lung cancer poor survival and cell cycle; (**B**, **C**) Genetic alteration in FAM72A-D and its association with OS of LUAD patients.

RNA-seq data obtained from TCGA were used to compare groups with high and low FAM72A–D expression. Approximately 20,000 differentially expressed genes were identified between the high and low expression groups. Based on a GSEA of all differentially expressed genes, various significantly enriched signaling pathways were identified, including EGFR signaling, lung cancer poor survival, undifferentiated cancer, proliferation, and cell cycle, as determined by the normalized enrichment score (NES), adjusted *P*-value, and false discovery rate (FDR) (Figure 7A–7D). Several pathways enriched in FAM72A–D were related to LUAD, including proliferation, EGFR signaling, undifferentiated cancer, lung cancer poor survival, and cell cycle pathways (Figure 8A).

### Genetic mutations in FAM72 and their associations with OS

We analyzed genetic alterations in *FAM72A–D* and their associations with OS in LUAD. As shown in Figure 8B, 8C, patients with LUAD showed a high *FAM72A–D* mutation rate. Among 510 patients with LUAD, genetic alterations were found in 122 patients, with an overall mutation rate of 24% and specific mutation rates of 14%, 17%, 12%, and 18% for the four loci, respectively. Furthermore, genetic alterations in *FAM72A–D* were associated with a poor OS in patients with LUAD. These results implied that mutations in *FAM72A–D* could affect prognosis in LUAD.

### Correlations between FAM72 expression and immune infiltration

As shown in Table 3, activated CD4 T cells were significantly positively correlated with FAM72A–D expression. In other subsets, we found that FAM72 expression was associated with plasmacytoid dendritic cell, natural killer T cell, monocyte, mast cell, macrophage, immature dendritic cell, eosinophil, activated dendritic cell, type 2 T helper cell, type 17 T helper cell, T follicular helper cell, memory B cell, immature B cell, gamma delta T cell, effector memory CD4 T cell, central memory CD4 T cell, activated CD4 T cell, and activated B cell counts. PDCD1 (PD-1) and CD274 (PD-L1) levels were positively correlated with FAM72A–D expression (Table 4).

**Table 3:** The association between the expression level of FAM72A-D and the immune infiltration in the tumor microenvironment.

**Immune cell**	**Spearman correlation**	***P*-value**
FAM72A		
Plasmacytoid dendritic cell	-0.275	<0.001
Natural killer cell	-0.134	0.002
Monocyte	-0.204	<0.001
Mast cell	-0.365	<0.001
Macrophage	-0.149	0.001
Immature dendritic cell	-0.184	<0.001
Eosinophil	-0.379	<0.001
Activated dendritic cell	-0.161	<0.001
Type 2 T helper cell	0.239	<0.001
Type 17 T helper cell	-0.221	<0.001
Type 1 T helper cell	-0.135	0.002
T follicular helper cell	-0.219	<0.001
Memory B cell	0.430	<0.001
Immature B cell	-0.134	0.003
Gamma delta T cell	0.106	0.017
Effector memory CD4 T cell	0.198	<0.001
Central memory CD4 T cell	-0.180	<0.001
Activated CD4 T cell	0.517	<0.001
Activated B cell	-0.222	<0.001
FAM72B		
Plasmacytoid dendritic cell	-0.225	<0.001
Natural killer T cell	-0.132	0.003
Monocyte	-0.185	<0.001
Mast cell	-0.347	<0.001
Macrophage	-0.114	0.010
Immature dendritic cell	-0.148	0.001
Eosinophil	-0.372	<0.001
Activated dendritic cell	-0.118	0.008
Type 2 T helper cell	0.297	<0.001
Type 17 T helper cell	-0.197	<0.001
T follicular helper cell	-0.190	<0.001
Memory B cell	0.484	<0.001
Immature B cell	-0.099	0.025
Gamma delta T cell	0.163	<0.001
Effector memory CD4 T cell	0.237	<0.001
Central memory CD4 T cell	-0.162	<0.001
Activated CD4 T cell	0.563	<0.001
Activated B cell	-0.188	<0.001
FAM72C		
Plasmacytoid dendritic cell	-0.295	<0.001
Natural killer cell	-0.145	0.001
Monocyte	-0.178	<0.001
Mast cell	-0.402	<0.001
Macrophage	-0.132	0.003
Immature dendritic cell	-0.209	<0.001
Eosinophil	-0.375	<0.001
Activated dendritic cell	-0.129	0.004
Type 2 T helper cell	0.177	<0.001
Type 17 T helper cell	-0.163	<0.001
Type 1 T helper cell	-0.119	0.007
T follicular helper cell	-0.228	<0.001
Memory B cell	0.335	<0.001
Immature B cell	-0.132	0.003
Effector memory CD4 T cell	0.156	<0.001
Central memory CD8 T cell	-0.106	0.017
Central memory CD4 T cell	-0.221	<0.001
Activated CD4 T cell	0.486	<0.001
Activated B cell	-0.153	0.001
FAM72D		
Plasmacytoid dendritic cell	-0.339	<0.001
Natural killer cell	-0.171	<0.001
Monocyte	-0.213	<0.001
Mast cell	-0.401	<0.001
Macrophage	-0.161	<0.001
Immature dendritic cell	-0.217	<0.001
Eosinophil	-0.379	<0.001
Activated dendritic cell	-0.179	<0.001
Type 2 T helper cell	0.253	<0.001
Type 17 T helper cell	-0.209	<0.001
Type 1 T helper cell	-0.159	<0.001
T follicular helper cell	-0.263	<0.001
Memory B cell	0.389	<0.001
Immature B cell	-0.166	<0.001
Gamma delta T cell	0.089	0.044
Effector memory CD4 T cell	0.140	0.002
Effector memory CD8 T cell	-0.108	0.015
Central memory CD4 T cell	-0.237	<0.001
Central memory CD8 T cell	-0.124	0.005
Activated CD4 T cell	0.523	<0.001
Activated B cell	-0.209	<0.001
MDSC	-0.098	0.001

**Table 4:** Correlation between the expression of PDCD1/CD274 and the expression of FAM72A-D.

	**Spearman correlation**	***P*-value**
PDCD1		
FAM72A	0.191	<0.001
FAM72B	0.211	<0.001
FAM72C	0.150	0.001
FAM72D	0.163	<0.001
CD274		
FAM72A	0.226	<0.001
FAM72B	0.248	<0.001
FAM72C	0.104	0.012
FAM72D	0.147	0.001

### Correlation between FAM72 expression and methylation

**Figure 9:**
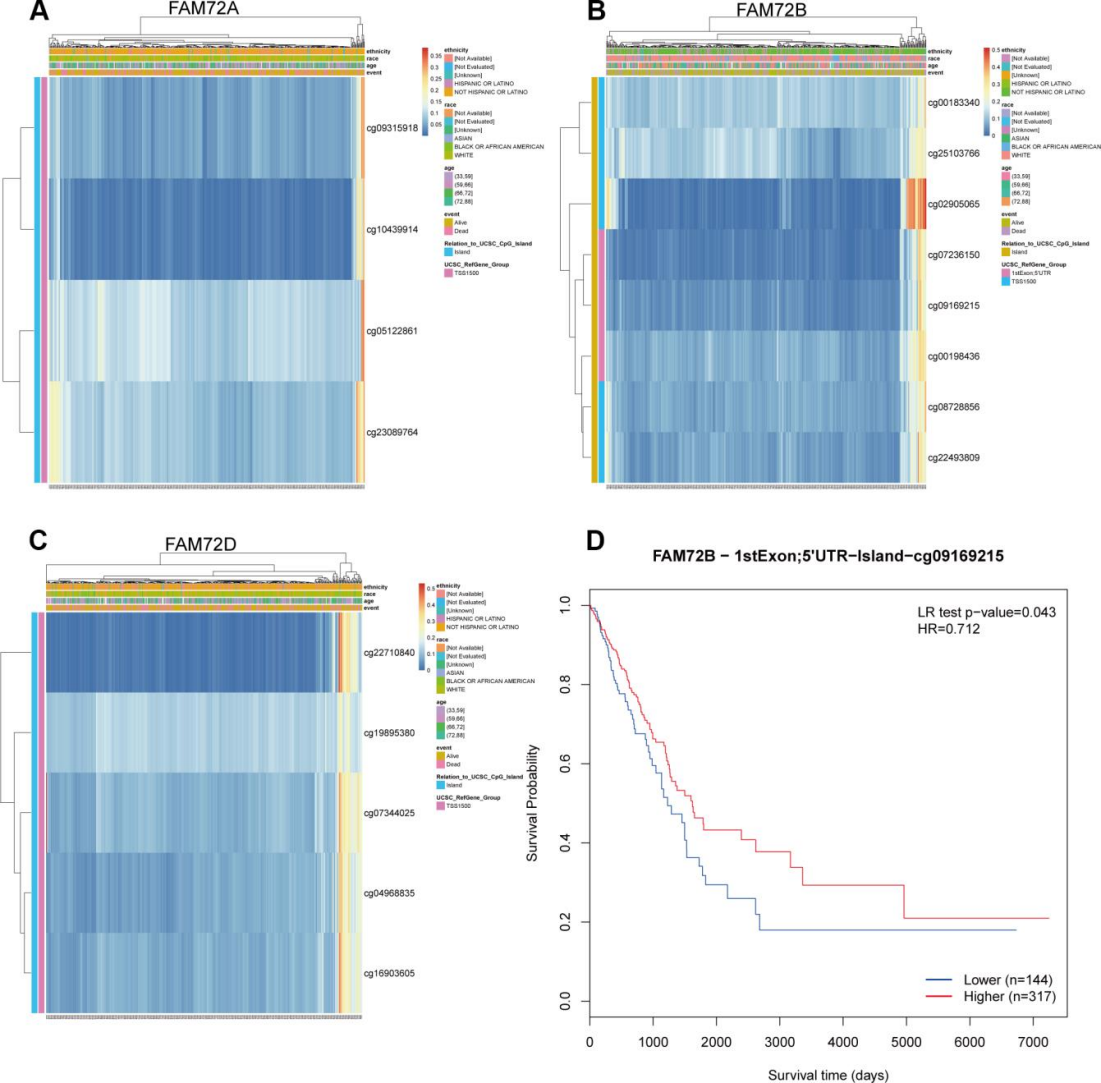
**The methylation of FAM72A/B/D in LUAD.** (**A**–**C**) The visualization between the methylation level and the FAM72A/B/D expression; (**D**) The Kaplan-Meier survival of the promoter methylation of FAM72B.

As shown in Figure 8A, the methylation level of FAM72 is low. Its expression may be related to the hypomethylation level (Figure 9A–9C). MethSurv was used to evaluate the effect of hypomethylation levels and FAM72 expression on prognosis in LUAD. We discovered that cg09169215, located in a CpG island, was associated with a poor prognosis (Figure 9D).

## DISCUSSION

Owing to the lack of early clinical features of LUAD, a large number of patients develop metastases [3, 4]. Currently, the primary clinical treatments for advanced LUAD are chemotherapy, radiotherapy, and targeted therapy; however, these approaches are limited with respect to improving survival. Moreover, the prognosis is poor for stage III or higher in NSCLC, with fewer than 15% of patients surviving 5 years [13]. Most patients eventually die from chemotherapy resistance and cancer progression. Therefore, the identification of new molecular mechanisms is necessary to develop therapeutic targets in LUAD.

Our results indicated that FAM72 may be a prognostic biomarker in LUAD. High FAM72 expression was associated with a poor survival rate. FAM72 mutations were also associated with poor survival. *FAM72* has been reported to promote cancer cell proliferation in glioblastoma and multiple myeloma [11, 12]. Rahane et al. indicated that the increased expression of mitotic FAM72 in tumor cells is caused by upstream mutations in primitive oncogenes or in tumor suppressor genes, such as *EGFR, RAS, BRAF,* and *TP53*, resulting in increased cell proliferation. Silencing neural stem cell-specific FAM72 may prevent cancer cell proliferation [11]. These previous findings suggest that *FAM72* is an oncogene.

Similarly, our results demonstrated *FAM72* was up-regulated in LUAD tissues, supporting its potential role in the development of LUAD. *FAM72* paralogs might contribute to tumorigenesis by the activation of centrosome and mitotic spindle formation via mitotic cell cycle genes *KIF23, ASPM, CEP55, KIF14, SGO1, CENPE,* and *BUB1* [11]. In addition, several studies have shown that FAM72 may be a target for tumor therapy [6, 10–12, 14]. However, the association between FAM72 and LUAD has not been explored to date. Therefore, our results provide the first evidence for its therapeutic and prognostic value in LUAD.

Key terms identified in a GO enrichment analysis, such as mitotic nuclear division, spindle, cell cycle and DNA replication, suggested that FAM72 is related to cell growth, division, and proliferation, further supporting its relationship with tumorigenesis. Other enriched terms were related to the centrosome, microtubule, and chromosome, and these findings need to be confirmed in further studies. A GSEA indicated roles in EGFR signaling, lung cancer poor survival, undifferentiated cancer, proliferation, and cell cycle pathways. Evan et al. reported that the excessive accumulation of cancer cells results from excessive cell proliferation and inadequate apoptosis. It is possible that FAM72 promotes tumorigenesis and contributes to a poor prognosis by similar mechanisms.

Immunotherapy is a promising approach in tumor therapy. The level of T cell immune infiltration is related to the efficacy of immunotherapy and is therefore the focus of current cancer immunotherapy research [15]. A large number of studies have shown that high levels of B cells and T cells, such as adenocarcinoma B cells and breast CD8T, are associated with a better OS in many types of cancer, including LUAD [16]. NK cells are important mediators of anti-tumor immunity and ultimately participate in malignant cell killing [17]. Our results showed that FAM72A–D expression levels are correlated with immune infiltration, including levels of T cells, B cells, eosinophils, NK cells, monocytes, mast cells, macrophages, and dendritic cells. These results suggest that the expression level of FAM72 may indicate the level of immune infiltration, providing a reference for the application of immunotherapy in LUAD. In addition, we found that PDCD1 (PD1) and CD274 (PD-L1) expression levels are positively correlated with FAM72A–D expression levels. High levels of PD-L1 have been detected in many tumors, including NSCLC, and are associated with poor prognosis [18]. However, some studies have indicated that elevated PD-L1 may be related to a better response to immunotherapy. A randomized phase 3 trial of pembrolizumab and docetaxel in patients with PD-L1-positive tumors suggested that the survival benefit was associated with PD-L1 expression (50% expression of PD-L1, HR = 0.53; 1–49% expression, HR = 0.76) [19]. Another study has shown that patients with PD-L1 overexpression have better clinical outcomes after anti-PD-1 therapy than those of patients with low expression [20]. Our results indicated that FAM72 is a candidate predictive biomarker for the efficacy of immunotherapy and is associated with prognosis in patients with LUAD receiving immunotherapy.

Several studies have demonstrated an inverse relationship between hypomethylation and gene expression in NSCLC [21, 22]. Sato et al. indicated that high gene expression induced by hypomethylation is associated with poor prognosis in NSCLC [23, 24]. Previous studies have shown that high FAM72 expression is associated with hypomethylation and outcomes in cancer [11, 12]. We found that FAM72A–D show hypomethylation and that the hypomethylation level is associated with a poor prognosis in LUAD. Therefore, FAM72 expression may be epigenetically regulated by DNA hypomethylation, providing an additional prognostic factor.

Although our findings improve our understanding of the relationship between FAM72A–D and LUAD, our study had some limitations. First, the data were obtained from public databases, and data quality cannot be assessed. Further experimental studies are needed to confirm our results. Second, the specific role of FAM72 in the development of LUAD should be comprehensively evaluated, and a broader range of clinical factors should be considered. These analyses were limited by a lack of some information in public databases. Third, the correlation between FAM72 expression at the mRNA and protein levels needs to be verified by cellular and clinical experiments. Fourth, we cannot determine the direct mechanisms by which FAM72 promotes the development of LUAD, which should be a focus of future research. To further investigate the mechanism underlying the effects of FAM72 in LUAD, we plan to conduct cellular experiments in the near future.

In summary, the results of the present study partially revealed the roles of FAM72A-D in LUAD, providing a potential therapeutic and prognostic biomarker. In particular, we found that FAM72 may be a molecular marker of poor prognosis in LUAD. *FAM72* mutations were associated with poor survival. Additionally, FAM72 may be a predictive biomarker for the efficacy of immunotherapy as well as prognosis in patients with LUAD undergoing immunotherapy. Finally, hypomethylation was associated with high FAM72 expression, providing additional insight into the molecular mechanisms underlying LUAD.

## MATERIALS AND METHODS

### Clinical information from TCGA and GEO databases

Gene expression data (HTSeq-Counts and HTSeq-FPKM), phenotype data, and detailed clinicopathological information for TCGA-LUAD were obtained using the UCSC Xena browser (version: 07-20-2019, https://xenabrowser.net/datapages/). Sequence data were retrieved using the Illumina HiSeq_RNA_Seq platform. HTSeq-FPKM gene expression data were transformed into TPM (transcripts per million reads) for subsequent analyses. TPM yields more similar results to those produced by a microarray approach and facilitates comparisons among samples [25]. The inclusion criteria for data retrieval from the two databases were as follows: (1) pathological diagnosis of adenocarcinoma, (2) complete gene expression data, and (3) complete survival information. GEO datasets used GPL570, GPL6480 and GPL6884 platforms. The probe ID was converted to the gene symbol according to the related annotation file, and the average expression values for multiple probes corresponding to the same gene were calculated. Data used in the study were in accordance with publication guidelines provided by TCGA and GEO. No studies directly involving human participants or animal experiments were included. Ethics approval and informed consent were not required.

### Statistical analysis

All statistical analysis was performed using the IBM SPSS statistical package (SPSS 26.0) and R (4.0.2). The Wilcoxon rank-sum test was used to compare the expression of FAM72A–D between LUAD and normal groups. The relationships between clinicopathologic parameters and FAM72A–D expression levels were evaluated with Wilcoxon signed-rank tests. Clinicopathologic parameters (age, gender, pack-years, race, tumor site, EGFR status, ALK status, KRAS status, T stage, N stage, M stage, pathological stage, and gene expression) associated with OS were evaluated by Cox regression analyses. Univariate and multivariate analyses were performed by applying the Cox logistic regression model to identify independent predictors, including age at diagnosis (ref. ≤70 years), gender (ref. male), pack-years (ref. <40), race (ref. other race), tumor site (ref. other site), EGFR status (ref. wt), ALK status (ref. wt), KRAS status (ref. wt), T stage (ref. T1), N stage (ref. NO), M stage (ref. M0), pathological stage (ref. I), and FAM72A–D expression (ref. Low). The 95% confidence interval (Cl) of the HR was measured to evaluate the hazard risk for individual factors. All tests were two-sided, and *P*-values of less than 0.05 were considered statistically significant.

### Over-expression of FAM72A-D in patients with LUAD

FAM72 expression levels in patients with LUAD were compared between tumor tissues and normal tissues. In addition, the corrplot R package (version:0.84, https://cran.r-project.org/web/packages/corrplot/index.html) was used to explore correlations between FAM72A, B, C, and D levels in LUAD. The AUC values for FAM72A–D were evaluated using pROC R package (version:1.16.2, https://cran.r-project.org/web/packages/pROC/index.html) and ggplot2 R package (version:3.3.2, https://cran.r-project.org/web/packages/ggplot2/index.html).

### Association between FAM72 and survival

A survival curve was generated using the survival R package (v. 0.1.3, https://cran.r-project.org/web/packages/survivalAnalysis/index.html) and survminer R package (v. 0.4.8, https://cran.r-project.org/web/packages/survminer/index.html). Furthermore, the prognostic value of the risk model was evaluated using four independent datasets from GEO (https://www.ncbi.nlm.nih.gov/geo/), GSE13213 (n = 117), GSE30219 (n = 85), GSE412710 (n = 181), and GSE50081 (n = 127).

### Construction and evaluation of the nomogram and prognostic model

A nomogram was constructed based on the optimal multivariate Cox regression analysis to predict the 1-year, 3-year, and 5-year survival probabilities. The rms R package (v. 6.0-1, https://cran.r-project.org/web/packages/rms/index.html) was used to produce a nomogram. The concordance index (C-index) and calibration plot are frequently used to evaluate the quality of nomogram models. The C-index and calibration plot were evaluated using the Hmisc R package (v. 4.4-1, https://cran.r-project.org/web/packages/Hmisc/index.html). In this study, the C-index was used to determine the discrimination ability with 1000 bootstrap replicated. As the C-index increases, the prediction accuracy increases. The calibration curve was visually evaluated by mapping the nomogram predictions to observed probabilities, and a 45° line represented optimal predictive values.

### Functional enrichment analysis

GEPIA, a database based on the UCSC Xena project (http://xena.ucsc.edu), enables dynamic analyses and visualization of TCGA gene expression profile data [26]. In our study, GEPIA2.0 (http://gepia2.cancer-pku.cn/) was used to find genes with highly correlated expression levels to those of FAM72A–D in LUAD. The correlations between levels of these genes and FAM72A–D were greater than 0.62. Then, the functions of FAM72A–D and genes with correlated expression in TCGA-LUAD were predicted by a GO enrichment analysis, as implemented using Metascape (http://metascape.org) [27].

### Gene set enrichment analysis

Expression profiles (HTSeq-Counts) were compared between high and low FAM72A–D expression groups to identify differentially expressed genes using the DESeq2 R package (v. 1.28.1, http://www.bioconductor.org/packages/release/bioc/html/DESeq2.html) in R (4.0.2). A GSEA of approximately 20,000 differentially expressed genes was performed. GSEA determines whether a set of prior defined genes show statistically significant and consistent differences between two biological states [28, 29]. In this study, GSEA was performed using the Molecular Signatures Database (MSigDB) Collection (c2.all.v7.0.entrez.gmt) of the clusterProfiler R package (3.18.0, http://bioconductor.org/packages/release/bioc/html/clusterProfiler.html) to identify statistically significant pathway differences between high and low FAM72A–D expression groups in LUAD. The expression level of FAM72A–D was used as a phenotype label. Pathway terms with adjusted *P*-value < 0.05 and FDR *q*-value < 0.25 were considered significantly enriched.

### FAM72A–D mutations and prognosis

cBioPortal (https://www.cbioportal.org) can be used to explore, visualize, and analyze multidimensional cancer genome data [30]. In our study, we analyzed the genomic profiles of FAM72A–D with a z-score threshold of ±1.8. Genetic mutations in FAM72A–D and their association with OS were evaluated.

### Analysis of immune infiltration with respect to FAM72A–D expression by ssGSEA

To evaluate correlations between FAM72A–D and levels of immune cell infiltration, the ssGSEA (single-sample Gene Set Enrichment Analysis) method was applied using the GSVA package (v. 1.36.3, http://www.bioconductor.org/packages/release/bioc/html/GSVA.html). The immune reference set was obtained from the literature (http://dx.doi.org/10.1016/j.celrep.2016.12.019) [31]. The relative levels of 28 types of tumor-infiltrating immune cells in the immunocyte signatures, including 782 genes for prediction in individual tissue samples, were evaluated. Based on 28 immunocyte signature genes in the literature, a relative enrichment score for every immunocyte was quantified from the gene expression profile of each tumor sample [32]. The following 28 types of immune cells were included: activated CD4 T cells, activated CD8 T cells, activated B cells, central memory CD4 T cells, central memory CD8 T cells, effector memory CD4 T cells, effector memory CD8 T cells, gamma delta T cells, regulatory T cells, type-1 T helper cells, type-2 T helper cells, type-17 T helper cells, follicular helper T cells, CD56 dim natural killer cells, CD56 bright natural killer cells, natural killer cells, natural killer T cells, immature B cells, memory B cells, activated dendritic cells, immature dendritic cells, plasmacytoid dendritic cells, mast cells, myeloid-derived suppressor cells, monocytes, eosinophils, neutrophils, and macrophages. The relationships between FAM72A–D and levels of immune cell infiltration were evaluated by Spearman correlation coefficients.

### Correlation between FAM72A–D expression and methylation

Studies have shown that FAM72 is related to methylation. Therefore, we further analyzed the relationship between FAM72A–D and methylation. Spearman correlation coefficients were determined to evaluate the correlation between FAM72A–D and methylation levels in TCGA-LUAD. For this analysis, MethSurv (https://biit.cs.ut.ee/methsurv/) was used, which is a web tool for univariate and multivariate survival analyses based on DNA methylation biomarkers using TCGA data, containing 25 different types of cancer and 7,358 patients [33].
